# Characterisation of the transcriptome of a wild great tit *Parus major *population by next generation sequencing

**DOI:** 10.1186/1471-2164-12-283

**Published:** 2011-06-02

**Authors:** Anna W Santure, Jake Gratten, Jim A Mossman, Ben C Sheldon, Jon Slate

**Affiliations:** 1 Department of Animal and Plant Sciences, University of Sheffield, Sheffield, S10 2TN, UK; 2 Edward Grey Institute, Department of Zoology, University of Oxford, Oxford, OX1 3PS, UK

## Abstract

**Background:**

The recent development of next generation sequencing technologies has made it possible to generate very large amounts of sequence data in species with little or no genome information. Combined with the large phenotypic databases available for wild and non-model species, these data will provide an unprecedented opportunity to "genomicise" ecological model organisms and establish the genetic basis of quantitative traits in natural populations.

**Results:**

This paper describes the sequencing, de novo assembly and analysis from the transcriptome of eight tissues of ten wild great tits. Approximately 4.6 million sequences and 1.4 billion bases of DNA were generated and assembled into 95,979 contigs, one third of which aligned with known *Taeniopygia guttata *(zebra finch) and *Gallus gallus *(chicken) transcripts. The majority (78%) of the remaining contigs aligned within or very close to regions of the zebra finch genome containing known genes, suggesting that they represented precursor mRNA rather than untranscribed genomic DNA. More than 35,000 single nucleotide polymorphisms and 10,000 microsatellite repeats were identified. Eleven percent of contigs were expressed in every tissue, while twenty one percent of contigs were expressed in only one tissue. The function of those contigs with strong evidence for tissue specific expression and contigs expressed in every tissue was inferred from the gene ontology (GO) terms associated with these contigs; heart and pancreas had the highest number of highly tissue specific GO terms (21.4% and 28.5% respectively).

**Conclusions:**

In summary, the transcriptomic data generated in this study will contribute towards efforts to assemble and annotate the great tit genome, as well as providing the markers required to perform gene mapping studies in wild populations.

## Background

The long-term study of wild vertebrate populations, whereby individuals are studied throughout their life histories, has provided enormous opportunity to understand a range of evolutionary and ecological questions [1]. Cavity-dwelling passerine birds have provided an ideal system for long-term study because they can be caught and handled easily, can be marked, tracked and measured from hatching to death, have short generation times and are sensitive to environmental change. A wealth of quantitative genetic studies have demonstrated a genetic basis for many of the life history traits commonly measured for passerines such as body size, timing of breeding and clutch size [2]. However, identifying the genomic regions which contribute to variation between individuals and between populations has, until very recently, been restricted by the lack of genetic resources in wild species [3].

The genomics era has provided an unprecedented explosion in the amount of sequence data available [4]. Recent advances in genomics, in particular next generation sequencing, mean that generating sequence data for non-model organisms has become accessible both in terms of timescale and price [5,6]. The integration of classical quantitative genetic studies with genomic data therefore provide an exciting opportunity for molecular ecologists to begin to understand the processes governing the evolution and maintenance of quantitative trait variation in wild populations.

The great tit (*Parus major*) is a small (~17 g) cavity nesting passerine species widespread and abundant in woodland across Eurasia. As they are highly amenable to using nest boxes, great tits have become a model study organism in behavioural ecology and evolutionary biology, particularly with regard to studying the evolutionary ecology of life history and morphological traits such as clutch size [7], lifetime fitness [8,9], fledgling mass and adult body mass [10-13]. More recently, quantitative genetic approaches have been extended to examine phenotypic plasticity in response to climate change [14-16], the heritability of personality traits [17] and reproductive senescence [18].

Wytham Woods, near Oxford, United Kingdom, has been the site of a continuous long-term study of great tits since the late 1940s, with nest boxes for great tits first erected in 1947 [19]. The birds have been intensively monitored, with basic morphological characteristics (adult body mass, adult wing length and fledgling mass), age, sampling origin within the woods and (social) pedigree relationships recorded for most individuals since the early 1960s. In addition, there is extensive information regarding life history traits such as breeding attempts, first egg date, egg mass, clutch size, hatching date, hatching success, fledging success, fledging size and recruitment success for those birds that recruit to the breeding population (see, for example, McCleery *et al*. [20] and references therein). More recent phenotyping projects have included measuring basal metabolic rate, personality [21], malarial infection and social network data for small subsets of individuals (Sheldon, unpublished data). Finally, blood has been sampled and stored for the majority of individuals alive since 2005, and for a subset of individuals alive between 1997 and 2005. The combination of extensive phenotypic data, pedigree information and blood samples (DNA) available from the Wytham Woods population provides tremendous opportunity to integrate the findings of previous 'classical' quantitative genetic studies of traits such as clutch size, body mass and personality in this species with quantitative trait locus (QTL) mapping to locate the regions of the genome contributing to variation in these traits [3]. Such mapping requires development of a set of markers spaced evenly across the genome. Next generation sequencing provides an efficient method for large-scale single nucleotide polymorphism (SNP) discovery [6,22-25], either by sequencing whole or partial genome (DNA) sequences or by sequencing transcript (cDNA) libraries generated from mRNA. Transcriptome sequencing (otherwise known as "RNA-seq") enables, in addition to SNP discovery, (i) the assembly and analysis of gene sequences likely to be contributing to key differences between individuals, (ii) analysis of tissue-specific gene expression, (iii) a greater depth of sequence coverage than would be possible from whole genome sequencing, (iv) the ability to infer the function of genes through comparative genomics and annotation, (v) the ability to find SNPs in known genes, including those that result in a change of protein, (vi) the ability to examine rates of molecular evolution in coding sequences, and (vii) the opportunity to test whether SNPs that explain phenotypic variation are in genes that show evidence of adaptive evolution between species. However, cDNA sequencing fails to cover regions of the genome that lacks genes, and because mRNA sequences are present in highly variable copy numbers within the cell, analysis is generally biased towards genes which are highly expressed. While normalisation of mRNA pools before sequencing enhances the probability of sequencing rare transcripts, including alternative splice variants, and gives the opportunity to infer elements of gene structure such as exon-intron boundaries, it restricts downstream analyses of transcript abundance. Transcriptome sequencing therefore complements whole or partial genome sequencing approaches.

Despite their prevalence in evolutionary and ecological studies, until recently the great tit had few genetic resources available, with only twenty two microsatellite markers cloned from the species and fewer than fifty full or partial gene sequences deposited in the NCBI nucleotide database http://www.ncbi.nlm.nih.gov/nuccore. However, in 2009 over two billion base pairs of genomic sequence was generated from ten great tits sampled from two populations in the Netherlands. The sequences were assembled into 550,000 contigs that cover an estimated 2.5% of the great tit genome [22]. In addition, the same research group has recently completed a low-coverage whole genome sequence of a single individual (van Bers, pers. comm). To complement this recently derived genomic sequence data, we have generated high read depth transcriptomic sequence data from the Wytham Woods population. In this paper, the sequencing, de novo assembly and analysis of the great tit transcriptome is described, highlighting the power and speed at which next generation sequencing enables the generation of sequence data in a wild species.

## Results

### Sequencing and Assembly

A total of 4,587,574 sequences with a combined length of 1.4 billion bases were sequenced. Read lengths averaged 302 bases, with a maximum length of 2,028 bases (Table 1). Pooling and trimming the sequences left a total of 4,130,014 reads for de novo assembly into contigs. The final NGen assembly ("the great tit assembly") yielded 95,979 contigs with four or more sequences, utilising 3,038,404 (74%) of the trimmed sequences. The mean contig length was 871 bases and the total length of assembled contigs was 83,601,386 bases. Despite normalisation of transcripts before sequencing, there was a large range (1-2,601) in the mean coverage depth of contigs (*i.e*., the mean number of reads in any given position in the contig), with overall mean coverage depth (accounting for difference in contig lengths) of 12.2x. The total number of sequences contributing to contigs was also highly variable, with a maximum of 9,633, and was relatively weakly correlated with the contig length (*r *= 0.45). The mean (standard error) and median number of sequences per contig was 32 (104) and 8 respectively. The 1,091,610 sequences not included in contigs ("singletons") had mean length 230 bases, making them shorter than the sequences included in contigs (323 bp) (*t *= 550.86, *p *<< 0.0001, two sample *t *test with unequal variance).

**Table 1:** 454 sequencing summary statistics

tissue	number of sequences	total base pairs sequenced	mean sequence length	maximum sequence length
brain	674,719	209,572,185	311	1,796
heart	408,547	112,462,495	275	1,863
kidney	655,004	198,351,347	303	1,256
liver	514,017	157,069,646	306	1,005
muscle	566,729	178,678,017	315	971
pancreas	532,017	174,131,123	327	1,726
skin	573,505	154,523,708	269	2,028
testes	663,036	199,038,872	300	1,737
all tissues	4,587,574	1,383,827,393	302	2,028

Most contigs were not expressed in every tissue (Table 2). 20,084 (21%) contigs were only expressed in one tissue (Table 2) and 10,335 (11%) contigs were expressed in all tissues. The remaining 68% were expressed in more than one, but not all tissues. Skin and testes seemed to have a relatively large number of highly tissue specific contigs (746 and 606 respectively).

**Table 2:** The total number of contigs expressed in the single tissue assemblies

tissue	total number of assembly contigs expressed	tissue specific contigs (number with τ ≥ 0.8)
brain	51,411	4,277 (212)
heart	58,988	939 (219)
kidney	44,367	2,223 (90)
liver	60,284	905 (89)
muscle	57,568	1,460 (221)
pancreas	58,098	1,441 (265)
skin	53,977	2,859 (746)
testes	44,463	5,980 (606)
total tissue specific contigs		20,084 (2,448)

Note that relatively few contigs were expressed at very high level in only one tissue (i.e. τ ≥ 0.8), the mean number of reads was 6 for contigs with τ < 0.8 and 25 for contigs with τ ≥ 0.8

### Transcriptome analysis

#### Blast against zebra finch, chicken and self

**Figure 1:**
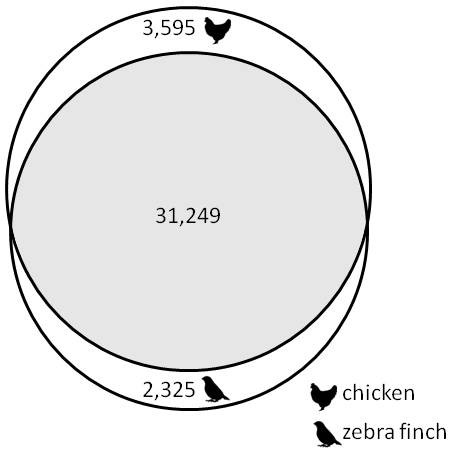
**Comparison of alignment of the 95,979 great tit contig sequences with the zebra finch and chicken cDNA databases**. Numbers represent the number of great tit contigs aligning to each database

Most contig sequences mapped to the zebra finch (97%) and chicken (67%) genomes but only around 1/3 aligned with sequences in the zebra finch and chicken transcriptome databases (Table 3). The divergence between chicken and passerines is thought to have occurred over 100 Mya while between zebra finch and great tit the divergence is ca. 50 Mya [26]. The great tit genome has 2n = 80 chromosomes [27] and, given the relative stability of avian chromosomes [[28], and references therein], it is expected that the great tit genome is similar in size (1.2 GB) to the zebra finch [29]. The majority of the contigs that aligned with zebra finch cDNA also aligned to chicken cDNA records (Figure 1). Only 1,913 (2%) contigs did not align with any of the zebra finch or chicken genome, cDNA or protein sequences; the mean length of these contigs was 512 bases so they were not unusually short. Approximately 54% of the sequences in the zebra finch cDNA database (18,597 sequences) and 49% of the sequences in the chicken cDNA database (22,314 sequences) matched great tit contigs suggesting that at least half of the previously known bird transcripts are represented in the assembled great tit contigs. The discordance between the number of contigs and the number of unique sequences they aligned with is due to multiple contigs mapping to the same gene. Given that the zebra finch cDNA database is 26.3 Mbp and the summed length of the reads contributing to contigs aligning with zebra finch cDNA sequences is 654.7 Mbp we estimate an overall transcriptome coverage of ~25×. This figure is lower than a predicted coverage of 41× (based on the amount of sequence generated) due to the low proportion of contigs aligning with zebra finch cDNA.

**Table 3:** Summary of blast searches between 95,979 great tit contigs and other avian sequence databases

zebra finch database			
	**whole genome**	**cDNA**	**protein**
	
contigs	93,221 (97%)	33,574 (35%)	29,830 (31%)
mean% similarity	90	95	90
mean coverage depth of contigs	12	17	17
unique hits	76,708	10,085	9,732

**chicken database**			

	**whole genome**	**cDNA**	**protein**
	
contigs	64,262 (67%)	34,844 (36%)	30,423 (32%)
mean% similarity	89	90	85
mean coverage depth of contigs	14	17	17
unique hits	51,161	10,943	10,480

The number of unique sequences in the zebra finch/chicken database (genome, cDNA or protein) that matched contigs ("unique hits") are also shown. Genomic DNA hits were not classified as unique if they were located less than 100 bases from any other hit

Of the ten most highly expressed contigs (mean coverage depth greater than 1000), six aligned to sequences in the zebra finch and/or chicken cDNA databases. These genes were Pleckstrin homology domain-containing family B member 2 (Golgi organisation and kinesin and protein binding), THO complex subunit 4 (RNA splicing), EF-hand domain-containing protein (calcium ion binding), Diamine acetyltransferase 1, Breast cancer anti-estrogen resistance protein 3 (cell adhesion) and Cadherin-11 Precursor (cell adhesion). Contigs mapping to zebra finch or chicken cDNA and with mean coverage depth greater than 100 are shown in Additional file 1, Table S1.

A small number of contigs aligned across most of their length to other contigs in the great tit assembly; blastclust grouped 525 contigs into 228 groups (between two to seven contigs per group) where there was similarity of 95% across all of the shortest contig. Relaxing the blastclust conditions slightly to a similarity of 95% across 95% of the shortest contig clustered 3,432 contigs into 1,184 groups (two to fifteen contigs per group). Given that the alignments are continuous across the shortest contig, there is no evidence to suggest that contigs within a cluster represent splice variants or that one or more of the contigs are pre-mRNA sequences. There is also little evidence to suggest that contigs within a cluster are expressed differentially in different tissues-for groups of two contigs the average correlation of tissue expression is 0.56 (182 pairs with 95% similarity across the shortest contig; for each pair the correlation was calculated from the total number of reads expressed in each tissue for each contig). Of the 182 pairs, 32 mapped to different genomic locations, suggesting that these contigs are paralogues. The remainder of contig pairs appear likely to represent different haplotypes of the same genomic region.

**Figure 2:**
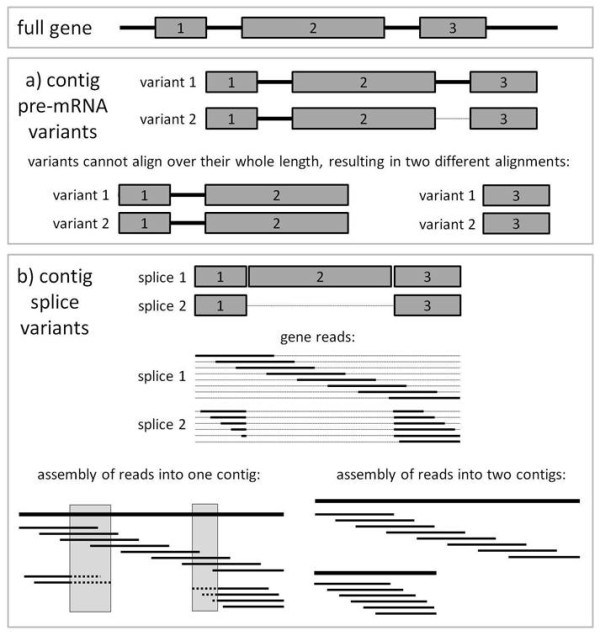
**Processes whereby reads from one gene can lead to complex contigs**. **a) Alignment with pre-mRNA sequences**. A gene sequence, including five and three prime untranslated regions, exons and introns, is initially transcribed completely from DNA into pre messenger RNA (pre-mRNA). Amplification and sequencing of RNA libraries may therefore generate contigs which represent pre-mRNA variants (some or all introns are included). In this example, variant 1 contains sequence from the three exons and both introns (solid lines) while variant 2 contains sequence from the three exons but only the first intron (solid line, the second intron is absent, represented by a thin dashed line). The same gene sequence may therefore be assembled into more than one contig. Alignment of such contigs is likely to occur across the whole length of one of the contigs, but occur disjointly for the other (in this case, the second intron in variant 1 cannot align to variant 2, so the two alignments between variant 1 and variant 2 occurs across the whole length of variant 2 but not variant 1) **b) Alignment and detection of splice variants**. Reads generated from two different splice variants are shown in the middle of the figure; reads "missing" an exon are discontinuous across the whole coding sequence. Bottom left: all reads are (incorrectly) assembled into a single contig, with reads from the second splice variant contributing to 'strings' of polymorphism (false SNPs) in the consensus sequence (regions shaded grey). Bottom right: reads from the two splice variants are assembled into separate contigs, with no regions of poor sequence similarity

Contigs were also blasted against themselves to identify contigs which did not necessarily share sequence homology across their entire length. A large number (~200,000) of reciprocal pairs of contigs were identified which aligned with e-value less than 10^-5^. Of these, a very large number (159,956) were single alignments, suggesting regions of shared protein domains across different genes. Five percent of single alignments extended over more than 95% of the shortest contig and, as expected, the set of single alignments included the 187 pairs identified by the blastclust analysis. Twenty-two percent (45,886) of reciprocal pairs of contigs had multiple alignments rather than a single alignment. In a sample of 200 pairs of contigs with multiple alignments, 93 aligned disjointly (Figure 2a), suggesting the two contigs may represent alternative splice variants, or one may be a pre-mRNA sequence. There were 25,589 contigs in one or more reciprocal pairs with multiple alignments, so a relatively large proportion (~12%) of the overall contig set may represent alternative mRNA or pre-mRNA sequences.

**Figure 3:**
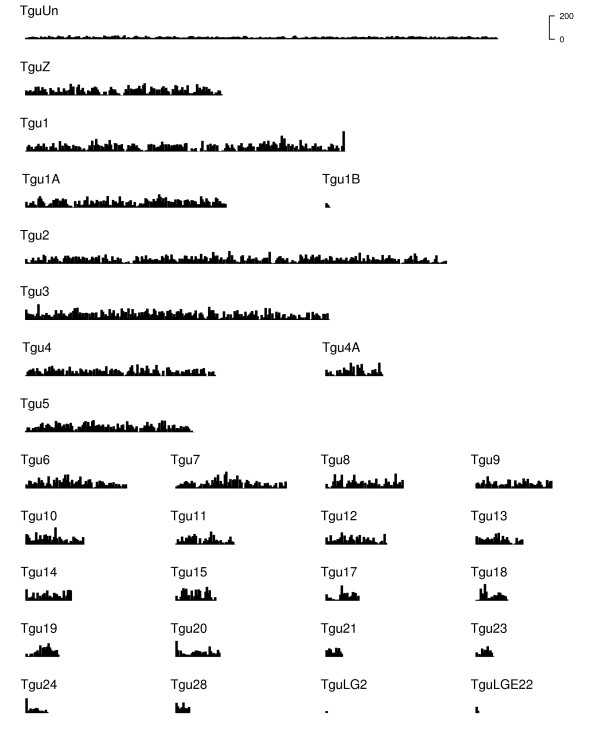
**Distribution of the number of contigs across the zebra finch genome**. Counts are plotted within 500,000 base pair bins. No contigs aligned with Tgu-16, 22, 25, 26 or 27, and one contig containing 17 reads aligned with TguLG5 (not shown)

**Figure 4:**
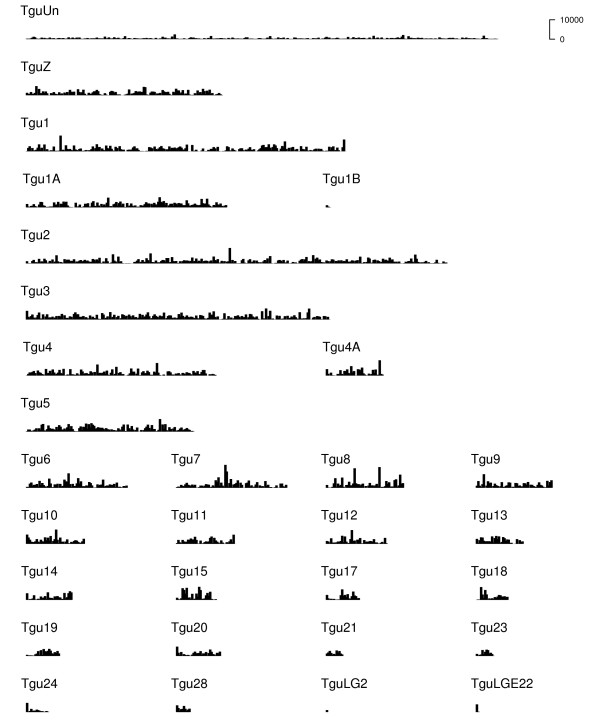
**Distribution of the total read depth of contigs across the zebra finch genome**. Counts are plotted within 500,000 base pair bins. No contigs aligned with Tgu-16, 22, 25, 26 or 27, and one contig containing 17 reads aligned with TguLG5 (not shown)

The overall contig distribution across zebra finch chromosomes is reasonably uniform across the genome (Figure 3), although some regions of very high expression are evident (Figure 4). There is a reasonable correlation between the distribution of contigs and the distribution of zebra finch genes across the genome (*R*^2 ^= 0.34; see Additional file 2, Figure S1 for the distribution of zebra finch genes across the genome). There was no relationship between chromosome length and contig density (*F*_1,28 _= 1.35, *p *= 0.26; *R*^2 ^= 0.04). The mean contig density across chromosomes (excluding TguUn) is 83 contigs per Mbp. Despite a higher gene density on zebra finch microchromosomes [30], there was no evidence to suggest that the density of contigs mapping to microchromosomes (Tgu-13-28 and Tgu1B)-and macrochromosomes (Tgu-1-5 and Tgu1A) differed (*t *= 0.84, *p *= 0.41, two sample *t*-test with unequal variance). No contigs aligned with chromosomes Tgu-16, 22, 25, 26 or 27, and one contig containing 17 reads aligned with TguLG5 (part of the assembly that remains unassigned to a chromosome). Tgu-16, 22, 25, 26, 27 and TguLG5 have a combined length of 1.4% of the genome and contain 4.1% of annotated zebra finch genes; it appears likely that by chance these chromosomes have remained unsampled.

The consensus great tit contig sequences contained 99.9% unambiguous bases (A, G, C or T) and 0.1% ambiguous bases (B, D, H, K, M, N, R, S, V, W or Y; these positions may either represent regions of poor sequence quality or possible single nucleotide polymorphisms). The mean GC content of the unambiguous bases in the great tit assembly is 40.7%. The mean GC content in the zebra finch and chicken cDNA databases is 50.4% and 48.6% respectively, while the mean genome GC content of the zebra finch and chicken genomes is 41.3% and 41.6% respectively. The GC content for regions of alignment between great tit contigs (45.4%) and zebra finch cDNA sequences (45.7%) are similar. However, extending the calculation across the whole contig and the whole zebra finch gene it aligns to gives mean GC contents of 42.6% and 48.2% respectively. These figures suggest that either the great tit has an unusually low GC content or that, if it is assumed that the great tit transcriptome has a similar GC content to zebra finch and chicken, the set of great tit contigs may contain genomic as well as transcriptomic sequence-i.e., that some contigs contain intronic or non-transcribed (i.e., pre-mRNA) sequences.

#### Gene Ontologies

The distribution of GO terms from the tissue-specific contigs into the three domains (biological process, molecular function and cellular component) is shown in Table 4. Note that in general the total number of GO terms is greater than the number of unique contigs, as many of the contigs map to zebra finch genes with more than one associated GO term. Of the 33,574 contigs aligning with zebra finch cDNA sequences, GO terms could be extracted for 20,847 (62%) of the contigs. There were 5,906 contigs with GO terms expressed in all tissues and 2,782 tissue-specific contigs with GO terms (Table 4). Many of the GO terms from tissue-specific contigs were found in more than one tissue (as many different genes may be associated with the same GO term). The tissue-unique GO terms (Table 4) are listed in Additional file 1, Table S2; a total of 114, 32, 58, 49, 44, 46, 67 and 117 GO terms were uniquely associated with contigs only expressed in brain, heart, kidney, liver, muscle, pancreas, skin and testes, respectively, while 1,654 GO terms were uniquely associated with contigs expressed in all tissues. A total of 84 tissue-unique GO terms were also highly tissue specific (τ > 0.8) (Additional file 1, Table S2). Interestingly, it was heart and pancreas that had the highest number of highly tissue specific GO terms (21.4% and 28.5% respectively), and of the seven terms with τ > 0.85, four were unique to heart (connexon complex, myosin filament assembly, A band and myosin heavy chain binding), two to pancreas (glucosamine 6-phosphate N-acetyltransferase activity and cholecystokinin receptor activity) and one to skin (proton-dependent oligopeptide secondary active transmembrane transporter activity). The tissue specificity of these GO terms generally appears biologically consistent, although it is interesting to note, for example, two "ovarian" GO terms associated with testes-ovarian cumulus expansion and ovulation cycle, and two "respiratory" GO terms associated with heart-bronchus morphogenesis and trachea formation (Additional file 1, Table S2). Such tissue overlap may reflect (incorrect) alignment of a contig to a protein which shares a conserved domain but has a different function.

**Table 4:** Summary of the assignment of GO domains to contigs

tissue	tissue specific contigs assigned with GO terms						number of GO terms
		
	number of contigs (number with τ ≥ 0.8)	mean τ	biological process	molecular function	cellular component	total GO terms	unique to that tissue (number with τ ≥ 0.8)
brain	517 (32)	0.73	1,020	327	262	1,609	114 (7)
heart	141 (43)	0.78	295	101	59	455	32 (18)
kidney	337 (18)	0.72	451	139	119	709	58 (8)
liver	219 (22)	0.75	260	114	56	430	49 (1)
muscle	265 (36)	0.75	307	75	70	452	44 (5)
pancreas	223 (42)	0.75	313	104	48	465	46 (24)
skin	433 (125)	0.77	593	126	114	833	67 (11)
testes	647 (54)	0.73	677	131	133	941	117 (10)
sum	2,782 (372)	0.75	3,916	1,117	861	5,894	527 (84)
all tissues	5,906	0.29	4,550	1,189	1,049	6,788	1,654

Contigs were assigned into the three GO domains (biological process, molecular function and cellular component) through 'is_a' pedigree mapping. The number of unique GO terms is also reported for each tissue

The three GO domains (biological process; GO:0008150, molecular function; GO:0003674 and cellular component; GO:0005575) have 65 immediate child terms (35 with parent 'biological process', 17 with parent 'molecular function' and 13 with parent 'cellular component'). Of the sixty-five terms, 21 were not represented in any of the 'is_a' mappings, two were associated with only one tissue (GO:0016247-channel regulator activity, associated only with brain, and GO:0005198-structural molecule activity, associated only with muscle), and the remaining 42 were all parents of unique GO terms expressed in all tissues (Additional file 1, Table S3). For combined 'is_a' and 'part_of' relationships, the same two GO terms were uniquely associated with brain and muscle, but only 14 were not represented in any of the "is_a" or "part_of" mappings, namely carbohydrate, carbon, nitrogen, phosphorus, sugar, and sulfur utilization (domain: biological process), chemoattractant, chemorepellent, electron carrier and nutrient reservoir activity and protein tag (domain: molecular function) and symplast, virion and virion part (domain: cellular component). The remaining 49 terms were expressed in all tissues (Additional file 1, Table S3)-i.e., seven GO terms mapped through only 'part_of' and not 'is_a' mappings-cell killing, cell wall organization or biogenesis, reproduction and viral reproduction (domain: biological process) and cell, extracellular region and extracellular region part (domain: cellular component) (Additional file 1, Table S3). These results indicate that although there is evidence for highly tissue specific expression of 2,448 contigs (Table 2), the GO annotations find only a few tissue-specific functions for these contigs (as classified by unique GO terms).

#### Mapping non-gene contigs

We further investigated the 59,955 contigs that mapped to the zebra finch genome but did not match anything in the zebra finch cDNA database. Approximately two thirds of contigs mapped either up or downstream of a gene (classes A and B, Table 5a, see Methods and Table 5 for a full description of classes A-F). Of the remainder, the vast majority (20,647) were class F, whereby the contig mapped entirely within the boundaries of the genome location of a known gene, although 49 contigs extended across the entire gene sequence (class E). There are similar numbers of contigs in classes A (gene upstream) and B (gene downstream), and approximately 30% of these contigs align to locations within 2,500 bases of a mapped gene (Table 5a). The distribution of the distance of class A and B contigs from their nearest gene differed significantly from the random distribution generated from 1,000,000 simulated contigs (p << 0.0001, test statistic = 0.31, Kolmogorov-Smirnov test, contigs mapping within genes excluded). In other words, Class A and Class B contigs are closer to genes than expected from the sequencing of random genomic DNA, implying that the majority of these "non-gene" contigs are either gene sequences or closely associated with gene sequences (for example involved in gene transcription or regulation) rather than erroneously amplified genomic sequence.

**Table 5:** Summary of the 59,955 contigs that aligned with regions of the zebra finch genome assembly, but had no match in the zebra finch cDNA database

a) classes A and B	total	distance to nearest gene (bp)				
		
		< 2,500	< 5,000	< 7,500	< 10,000	
	19,357	5,843	8,429	9,778	10,752	
	19,721	5,708	8,227	9,621	10,570	

**b) classes C^1 ^and D^2^**	**total**	**alignment position of contig**				

		**start position**				
		
	84	**penultimate intron**	**final intron**	**final exon**	**3' UTR**	
		
		1	7	74	2	
		
		**end position**				
		
	97	**first exon**		**first intron**		
		
		86		11		

**c) class E^3^**	**total**	**gene length (bp)**		**length of alignment with** **genome sequence (bp)**		

	49	64-202		131-1,235		

**d) class F^4^**	**total**	**aligns to one gene feature^5^**		**aligns to more than** **one gene feature^6^**		

		20,387		260		
		
		**one gene feature^5^, contig aligns to**				
		
		**5'UTR**	**3'UTR**	**exons**	**introns**	
		
	20,647	4	89	4	20,290	
		
		**more than one gene feature^6^, contig aligns to**				
		
		**contig start**		**contig end**		
		
		**exons**	**introns**	**exons**	**introns**	**3' UTR**
		
		111	149	105	152	3

Contigs are shown in grey while genes are in black

^1 ^class C contigs align to genes with a mean of 10.5 exons (range 1-35 exons), 18% of genes have only one exon

^2 ^class D contigs align to genes with a mean of 8.4 exons (range 1-33 exons), 20% of genes have only one exon

^3 ^48 contigs extend from 5' (upstream) to 3' (downstream) of the closest mapped gene and one contig extends from the start position (i.e., the start of the 5'UTR) of the closest gene to 3' of the gene. All genes have only one exon

^4 ^class F contigs align to genes with a mean of 16.4 exons (range 1-149 exons), 87% of genes have more than five exons

^5 ^for example, the start and end of the contig are both within the 5' UTR

^6 ^for example, the start of the contig aligns within exon 1 and the end of the contig aligns within intron 1

A small number of Class A and B contigs aligned with non-coding RNA sequences. Of the 700 non coding RNA sequences described in the zebra finch genome, including micro, ribosomal, small nucleolar and small nuclear RNA, 45 contigs aligned to 27 unique non-coding RNA sequences. Twenty two of the non-coding RNA contigs mapped within 2,000 bases of a gene, suggesting that these non-coding RNAs may be involved in modulating the expression of the nearby gene. Only ten of the 27 non-coding RNAs have an associated gene name and description, six of which are small nucleolar RNAs. However, it is likely that there are many more than 700 non-coding RNA sequences in the zebra finch (and, indeed, great tit) genomes; 4,431 have been described in zebrafish (*Danio rerio*) and 8,383 in human (assembly and gene build genome statistics, http://www.ensembl.org). Thus, at least a few hundred of the class A and B contigs may represent non-coding RNA.

**Figure 5:**
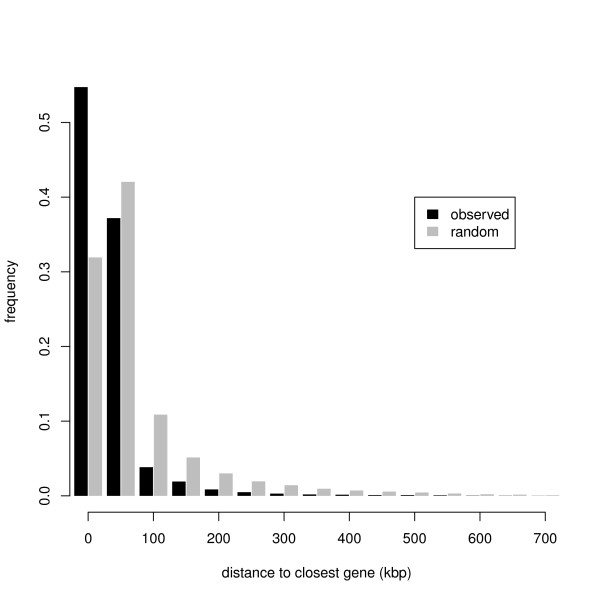
**Distribution of the predicted minimum distance between known genes and contigs randomly placed on the zebra finch genome (estimated from 1,000,000 simulated contigs), and the observed minimum distance between known genes and alignments with contigs in the great tit assembly (85,109 contigs)**. Note that ~8,000 contigs were excluded as they map to unassembled regions of the zebra finch genome (for example, Tgu 1_random)

The distribution of the distance between all contigs mapping to the zebra finch genome (including those mapping within genes) also differed significantly from the distribution expected at random (p << 0.0001, test statistic = 0.34, Kolmogorov-Smirnov test, Figure 5).

For most class C contigs, alignments begin in the final exon of the gene, while for most class D contigs alignments end in the first exon (Table 5b). All genes enclosed by class E contigs contain a single exon and tend to be small (range 64-202 bases, mean 94 bases, Table 5c). In class F, the vast majority (20,290) of contigs aligned within a single intron (see 'one gene feature' in Table 5d). Two hundred and sixty contigs aligned across more than one gene feature, with 57% of alignments beginning in an intron, and 58% of alignments ending in an intron (see 'more than one gene feature' in Table 5d). Thus, the overwhelming majority of class F contigs align with intronic regions of a zebra finch gene, providing very strong support that these contigs represent pre-mRNA (i.e., the RNA transcript has not been spliced to form mRNA). The mean number of reads per contig for the 20,566 class C, D, E and F contigs which align to intronic regions was 14; this differed significantly (*t *= 35.59, *p *<< 0.0001, two sided *t *test with unequal variance), from the mean (52 reads) for the 33,574 contigs which aligned with sequences in the zebra finch cDNA database (Table 3). Therefore pre-mRNAs are much less abundant than mRNA sequences.

#### Metagenomics of non-great tit sequences

Of the 1,913 contigs which did not align with any zebra finch or chicken genome, cDNA or protein sequences, 80 aligned to RefSeq RNA sequences. Of these, 17 aligned to vertebrate_birds, suggesting they are likely to represent true great tit sequences. Of the remaining 63 contigs that did not align to vertebrate_birds, 22 aligned to another vertebrate group (vertebrate_fish, vertebrate_amphibians, vertebrate_mammalian or vertebrate_reptiles), suggesting that these contigs represent great tit genes without an annotated vertebrate_bird homologue in the RefSeq RNA database. A further 26 contigs aligned with species from two or more kingdoms, and alignments were typically short (< 100 bases) or did not span more than 20% of the contig sequence. Of the remaining 15 contigs which aligned with species from a single kingdom, one aligned with bacterial species, and 14 with plant species. The 'bacterial' contig aligned with 57 different bacterial genera. The remaining 14 contigs aligned with *Populus trichocarpa *(twelve contigs), or both *P. trichocarpa *and *Sorghum bicolor *(two contigs) sequences, and appear most likely to represent non-great tit sequences. The two *S. bicolor *alignments and two of the *P. trichocarpa *alignments were shorter than 80 bases and covered less than 12% of the contig, suggesting the alignments are unlikely to represent *Populus *or *Sorghum *but may be plant contamination of some kind. Note that while *P. trichocarpa *and *S. bicolor *are model species with full genome (and transcriptome) sequences, sequences from trees common in the ecology of the great tit (oak, ash, beech, hazel, sycamore) are not well represented in the RefSeq RNA database. Eleven of the remaining twelve contigs were assembled from skin reads, and the other from a combination of skin and testes reads. The remaining alignments with *P. trichocarpa *were of reasonable length (109-438 bases) and identity (84-94%), suggesting there may be sequences from a plant species related to *P. trichocarpa *in the great tit assembly (oak, beech and hazel are more closely related to *P. trichocarpa *than ash or sycamore).

Alignments with 'environmental' sequences (i.e., sequences from species important in the ecology of the great tit, see Methods) provide some evidence to suggest that the great tit assembly includes sequences from flea and tick (ectoparasites that commonly inhabit bird nests). Forty-six contigs aligned with ribosomal RNA sequences from *Ixodes ricinus*. Twenty-eight contigs aligned with ribosomal RNA sequences from *Ceratophyllus ciliatus *(a grey squirrel flea), with some but not all of these contigs also aligning with sequences from the hen flea *Ceratophyllus gallinae*. However, contigs aligning to *Ceratophyllus ciliatus *and to *Ixodes ricinus *showed no evidence of tissue specificity; all ten of the most significant contig alignments with *C*. *ciliatus *were expressed in two or more tissues. Similarly all ten of the most significant contig alignments with *I*. *ricinus *were expressed in two or more tissues. Of the 1,091,610 singletons not assembled into contig sequences, 3,656 aligned with 60 'environmental' sequences. Alignments with flea (*Ceratophyllus *spp) and tick (*Ixodes ricinus*) sequences were the most prevalent (1,524 and 829 alignments), in addition there were 700 alignments with badger (*Meles meles*)-hair from which is frequently incorporated into nests-and 364 with beech (*Fagus sylvatica*). Thus, there is limited evidence that small amounts of RNA from other species have been included in the great tit sequences. There was little evidence to suggest that the great tit assembly includes sequences from any common endoparasites, nor from the great tit's common food source the winter moth (*Opheroptera brumata*).

#### Microsatellite repeat discovery

SPUTNIK detected 10,272 repeats of between two and five bases with five or more repeat units in 8,514 contigs (Table 6). The total sequence length of di-, tri-, tetra-and penta-nucleotide repeats with more than five repeat units represents approximately 0.2% of the total assembled contig sequences. Fifty-five of the repeats matched (with an e-value of 10^-10 ^or less) 46 passerine microsatellites isolated from 25 different species. All di-, tri-, tetra-and penta-nucleotides with at least 5 repeat units, along with up to 200 bases total flanking sequence, are listed in Additional file 1, Table S4.

**Table 6:** The distribution of microsatellite repeat sizes and lengths

repeat type	number of repeat units				maximum repeat units	total
			
	5-9	10-14	15-19	> 19		
dinucleotide	5505	424	61	35	262	6,025
trinucleotide	2593	114	11	15	131	2,733
tetranucleotide	1065	59	19	23	130	1,166
pentanucleotide	304	20	6	18	66	348
all	9467	617	97	91		10,272

#### SNP discovery

Of an initial 1,038,094 SNPs identified by SeqMan NGen, 23,831 'high' and 35,579 'modest' quality SNPs were detected in 13,026 and 16,187 contigs respectively. The majority of the loss of SNPs was due to exclusion of SNPs with a minor allele count of less than three (~930,000 SNPs). Of the filtered SNPs, 22,427 (94%) high and 33,185 (93%) modest quality SNPs were confirmed by re-assembling pooled and trimmed sequences onto the consensus great tit assembly sequences. Although 35% of all great tit contigs align with zebra finch cDNA (Table 3, Figure 1), 46% of contigs containing high quality SNPs and 49% of contigs containing modest quality SNPs aligned with zebra finch cDNA. The reading frame of 4,822 high and 8,194 modest quality SNPs could be inferred from regions of alignment between zebra finch genes and the SNP-containing contig. Of these, 1,503 (31%) of high and 3,541 (43%) of modest quality SNPs caused non-synonymous substitutions while the remainder were not predicted to cause a change in the amino acid sequence (i.e., are synonymous substitutions). Both the number of SNPs and the number of microsatellites mapping to zebra finch chromosomes were highly correlated with the total length of these chromosomes (*r *> 0.98), and the overall distribution of microsatellites and SNPs is reasonably uniform across the genome (data not shown).

#### Alternative splicing

Three hundred and thirty-two contigs (0.3%) contained 'strings' of at least six SNPs, suggesting these contigs are likely to include reads from alternative splice variants (strings of SNPs suggest regions of poor alignment representative of alternative splicing, see Methods). Of these, 195 contained only one SNP string. All but 13 of these contigs aligned with the zebra finch genome, and 187 aligned to a mapped gene. Contigs containing SNP strings were expressed in more tissues than contigs without SNP strings (difference of 3.07 tissues, *t *= 64.14, *p *<< 0.0001, two sided *t *test with unequal variance) and were also less tissue specific (that is, were not predominantly expressed in only one tissue) than contigs without SNP strings (τ difference of 0.29, *t *= 20.62, *p *<< 0.0001). This difference was smaller but still significant once contig length and the number of reads mapping to the contig was taken into account (difference of 0.14 tissues, generalised linear model with Poisson distributed errors, *z *= 13.34, *p *<< 0.0001). Although two of the alternative splice contigs were expressed in only one tissue (one each in heart and liver), two hundred and sixty two (79%) of the 332 alternative splice contigs were expressed in all eight tissues. These results suggest that it is uncommon (< 1%) for a single tissue to generate more than one splice variant, and that for those alternative splice contigs expressed in more than one tissue, different tissues may be generating different alternative splice variants.

## Discussion

The sequencing of the great tit transcriptome represents an exciting application of next generation sequencing technology to the genome of a species which, until recently [22], had very few genetic resources. Although the downstream analysis of the great tit assembly was vastly enhanced by the availability of both zebra finch (*Taeniopygia guttata*) [30] and chicken (*Gallus gallus*) [31] complete genome sequences, the assembly of 454 reads was possible without a reference genome (i.e., de novo assembly) due to both the length of sequences (mean ~300 bases) and the high coverage (25×). The resultant great tit transcriptome assembly of 95,979 contigs, encompassing 85,997,184 bases, is a valuable tool for future genomic work in this and other closely related species, in particular for molecular evolution studies [32] and to enable the development of a panel of markers for QTL mapping and genome wide association studies. This illustrates the power of next generation sequencing to begin to illuminate the genetic architecture and evolution of quantitative traits at a genomic level in non-model and wild species.

As illustrated by the number of high quality SNPs detected, transcriptome or genomic sequencing is also a rapid method for detecting variation in a population. In addition to SNPs, the microsatellites identified in the transcriptome are a useful resource, as they provide new markers for this species. By seeing which of these markers are also found in the zebra finch assembly, it should be possible to determine which are the most conserved microsatellites in passerines. Such conserved microsatellites provide a good system for studying microsatellite evolution without any biases due to them being cloned in one species. Finally, because the microsatellites documented here are in or close to genes (given that the majority of contigs either align with genes (Figure 2) or map to within 5,000 bp of a gene (Table 5)), they are ideal markers for candidate gene studies or other population genetic studies requiring a modest number of variable markers which are uniformly distributed across the genome.

Mapping the great tit contigs to the zebra finch and chicken cDNA databases resulted in around one third of contigs aligning with known genes, despite almost all contigs aligning to the zebra finch and chicken genomes (Table 3). Given that all sequences were sequenced from complementary DNA, it seems surprising that a larger proportion of contigs did not match known genes. A number of possible explanations why some contigs did not map to known genes include: (i) the contigs are great tit genes without homologues in zebra finch or chicken, (ii) they are contigs of great tit genes which are highly divergent from their zebra finch and chicken homologues and therefore do not match in a blast search, (iii) they are non-great tit sequences, (iv) they are from genes which are unannotated in both zebra finch and chicken and (v) they are sequences of preprocessed mRNA (pre-mRNA).

It is very possible that a small number of contigs are either great-tit specific genes (i) or have undergone substantial sequence evolution since the divergence of zebra finch and great tit 40-45 million years ago (ii), but this explanation cannot account for most of the contigs that did not match the cDNA databases, because these contigs still matched genomic sequences. For the same reason, it appears highly unlikely that a substantial proportion of the contigs represent non-great tit sequences (iii). Given the comprehensive annotation of the chicken genome in particular, it also seems improbable that many contigs are as-yet unidentified genes (iv). The most intriguing explanation is that a high proportion of the non-gene contigs represent pre-mRNA (v). Of the contigs which did not align with zebra finch genes but did align with regions of the genome, many aligned to regions very close to genes. Further, many of the 'non-cDNA' contigs aligned within intronic regions, providing strong support that these contigs encode pre-mRNA rather than mRNA sequences.

Normalisation of the mRNA library prior to cDNA synthesis and sequencing is likely to have contributed to the presence of pre-mRNA sequences in the great tit assembly, due to the amplification of low copy number transcripts. The read depth of contigs identified as likely pre-mRNA transcripts is substantially lower than the read depth for contigs aligning with zebra finch genes (cDNA), suggesting that without normalisation the difference would be even larger. Thus, pre-mRNA sequences are less likely to be sequenced (and assembled into contigs) in non-normalised studies. However, the ability to align contigs to an assembled genome sequence (either the species itself or one closely related, in this case, zebra finch) and use this information to annotate contigs as mRNA, pre-mRNA and alternatively spliced transcripts provides a valuable tool. Even in the absence of an assembled genome to align to, it may be possible to align contigs with each other and infer elements of gene structure such as exon-intron boundaries (see Figure 2). Indeed, the presence of contigs covering non-coding regions may actually offer some advantages for downstream applications. In particular, it is now possible to type SNPs with different properties-for example, SNPs which map to intronic, 5' UTR, 3' UTR, and intergenic regions are valuable "neutral" contrasts to synonymous or non-synonymous SNPs located within coding regions, which are likely to be under stronger selection.

The high number of pairs of contigs with multiple blast alignments, compared to the number of contigs with SNP strings, suggests that the assembly software usually splits splice variants into discrete contigs, rather than combining sequences from multiple splice variants into one contig (Figure 2b). Our results may underestimate the overall number of alternatively spliced genes in the transcriptome; recent estimates suggest that at least half of all mammalian genes with more than one exon may be alternatively spliced [33,34]. One explanation for the small proportion of contigs identified as potential alternatively spliced genes is that contigs do not necessarily cover the total gene sequence, and different contigs may map to different parts of the same gene. Thus, signals of alternative splicing such as SNP 'strings' may be relatively rare given the overall coverage of the transcriptome. On the other hand, a large number of sequences (> 1 million) were not assembled; it may also be possible that some of these sequences represent minor splice variants which have not been sequenced with sufficient depth to have been assembled into contigs. An exciting area for future research would be to examine splice variants in more detail; for example, looking to see if different types of genes (e.g. immune genes) have evolved to have splice variants more often than others.

The 454 sequences from each of the eight tissues are deposited in the Sequence Read Archive (SRA), reference SRA026719.

## Conclusions

By describing and mining the transcriptome of a wild bird population we have facilitated future evolutionary genomics studies in this species. Genome-wide association studies, using SNPs in known genes, will pave the way for QTL discovery and insight into microevolutionary processes. Similarly, comparison of great tit coding regions with homologous regions in other sequenced bird species (e.g. great tit, zebra finch, turkey) will make it possible to identify genes with elevated rates of molecular evolution. By combining and integrating data from these two discrete types of analysis, it will be possible to investigate whether the same genes are relevant to micro and macro-evolutionary processes. In summary, next generation sequencing of the great tit transcriptome has paved the way to convert a classical ecological organism into one with a genetic toolkit.

## Methods

### Sequencing

Ten unrelated great tit nestlings, at 14 days post hatching, were sampled from across the Wytham Woods population and brain, heart, kidney, liver, muscle (pectoralis major), pancreas, skin and testes/ovaries dissected from each individual at between three and 23 minutes after death. Each chick was sexed at sampling and sex was later confirmed molecularly with the Z-002A marker [35]. Tissues were stored in RNALater (Ambion) to prevent RNA degradation. RNA was extracted using the TRIzol^® ^(Invitrogen) method. RNA quantity and degradation was tested using a Nanodrop (Agilent) with each tissue yielding a mean (standard error) of 44.4 (7.5) μg of RNA with mean (standard error) RNA integrity number (RIN) of 8.37 (0.21). For each tissue 1 μg of RNA from each bird was pooled, i.e. to make 8 tissue pools, each containing 10 μg of RNA. Complementary DNA (cDNA) was synthesised by Evrogen (Moscow, Russia) from the eight tissue-specific pools using SMART kits [36]. cDNA was normalised, also by Evrogen. cDNA sequencing was performed by the Liverpool Advanced Genomics Facility, with 3 μg of normalised cDNA sequenced on a 454 FLX Titanium Genome Sequencer. Each tissue was sequenced on half a plate, i.e. four plates were sequenced in total, giving an expected 1600 Mbp of sequence (mean read length 400, 1 M reads per plate; http://454.com). The zebra finch transcriptome is 26.3 Mbp; assuming that ~68% [37] of the total sequence meets quality criteria and assembles into contigs, a transcriptome coverage of ~41 is estimated.

### Assembly

Our strategy for transcriptome assembly was to first combine sequences from all tissues to create a de novo assembly, and then map sequences from each tissue onto the assembly to examine tissue-specific expression. Using the program NGen (version 2) (DNASTAR, Inc), sequences within each tissue pool were combined and screened to remove poly(A) tails, SMART kit primer sequences (added during cDNA library construction), adaptor sequences (added to enable purification, amplification and sequencing by the FLX technology) and poor quality sequences (average sequence quality less than 14 in a 30 base window). The trimmed sequences were then assembled de novo (i.e., assembled without a reference) with NGen after first assessing the impact of varying various assembly parameters. The final parameter settings of match size 41 (default 19), match percentage 90 (default 85), mismatch penalty 25, gap penalty 25 and minimum average sequence quality 14 were chosen on the basis that they (i) obtained coverage most similar to our expectation of 41x, (ii) had strict settings for match size and minimum match percentage, and should therefore be conservative and (iii) produced the overall highest mean contig length and number of sequences per contig. The choice of assembly was verified by visual examination of the quality of the alignments within a random subset of contigs. For all assemblies, contigs with less than four sequences were excluded from further analysis.

Tissue-specific assemblies were also made. Following removal of poly(A) tails, SMART kit primer sequences, adaptor sequences and poor quality sequences with NGen, each of the eight tissue pools was separately mapped onto the all-tissues transcriptome assembly. Default parameters for a reference-guided assembly were used, except for a longer match size (41, default 19) and higher match percentage (90, default 85) than the default settings.

#### Tissue specificity

Potentially some contigs may be expressed in all eight tissues, and some in only one tissue. For each tissue assembly, we assessed how many sequences were assembled into each contig (i.e. by examining the read depth of the contig). Contigs which were absent from a tissue-specific assembly were regarded as not expressed in that tissue at the time of sampling. Contigs which are expressed in all tissues (i.e. each tissue contributes at least one sequence read to the contig) and contigs which were tissue specific (all reads come from a single tissue, with a minimum of four reads from that tissue) were identified. In addition, more formally for each contig a measure of tissue specificity (τ) was calculated. Values for τ range from 0 (transcripts equally expressed in all tissues) to 1 (transcript expression is highly tissue-specific, i.e. very high expression in one tissue). τ accounts for differences in the total number of reads in different tissues [38], and was calculated following the approach of Mank *et al*. [39] to account for small levels of undetected expression;(1)

where *N *is the number of tissues and TPM_max _is the highest level of expression of a given contig over all *i *tissues examined. Expression for each contig is standardised to number of reads per million (TPM), i.e. the number of reads contributing to the contig, per million reads expressed in the whole tissue;(2)

where library*_i _*is the total number of reads expressed in library *i*. To reduce sampling stochasticity, TPM*_i _*is set to 2 for values of TPM_max _and reads*_i _*close to 0 [39].

### Transcriptome analysis

#### Blast against zebra finch, chicken and self

To estimate transcriptome coverage and to identify genes which have been sequenced, the great tit contigs were aligned with *Gallus gallus *(chicken/red junglefowl) and *Taeniopygia guttata *(zebra finch) sequences. The latest *Taeniopygia guttata *(version 3.2.4.58) and *Gallus gallus *(version 2.58) genome (DNA), predicted gene (cDNA) and predicted peptide (protein) databases were downloaded from the Ensembl ftp site http://www.ensembl.org/info/data/ftp/index.html. The great tit contigs were blasted against each database using stand-alone BLAST (version 2.2.22 for 64 bit Windows). The blastn program was used for searching against DNA and cDNA databases and blastx against peptide databases, with a cut-off expectation value (e-value) of 10^-5 ^and all other settings as default. The best alignment was retained for each contig.

To examine whether the assembly contained contigs with high sequence similarity (which could be suggestive of sequences from the same gene being assembled into more than one contig), contigs were clustered using blastclust http://www.ncbi.nlm.nih.gov/staff/tao/URLAPI/blastall with options-S 95.0-b F-p F-W 30 and -L 0.95 or 1.0, the latter option specifying alignments across either 95% or the whole length of the shortest contig. Contigs were also blasted against each other, with a cutoff e-value of 10^-5^. Contig pairs with a single alignment which does not extend over the full length of the shortest contig may represent different genes which share a conserved protein domain. In contrast, multiple alignments between two contigs may indicate that the contigs represent different splice variants of the same gene, or that one contig is an incompletely processed form of the mRNA sequence containing introns and other features not present in the final mRNA molecule (i.e., pre-mRNA) while the other contig is the final mRNA (Figure 2a).

#### Gene Ontologies

Gene Ontology (GO) terms are available for a large number of zebra finch genes, providing the opportunity to infer the function of those contigs in the great tit assembly which align with zebra finch genes. The latest Ensembl *T. guttata *(version 3.2.4.58) gene database was downloaded from the Ensembl website http://www.ensembl.org/index.html using the MartView interface of the BioMart data management system http://www.biomart.org. The Gene Ontology full ontology relationship file was downloaded from the Gene Ontology website (OBO version 1.2; http://www.geneontology.org/GO.downloads.ontology.shtml). Using the full ontology file and the outputs from blasting the great tit assembly against the *T. guttata *cDNA database, GO terms for each great tit contig were inferred. Note that 74% of the 18,191 zebra finch unique transcript identification numbers downloaded from Ensembl have been assigned GO terms, so GO terms could not be assigned to all contigs.

For contigs previously identified as tissue-specific or expressed in all tissues (see "Tissue specificity" above), tissue-unique GO terms were identified by finding GO terms associated with tissue-unique contigs which were not associated with contigs from any other tissue. To investigate whether individual tissues were associated with specific GO terms, the 'pedigree' of each of these tissue-unique GO terms was determined from the GO full ontology file. All GO terms are assigned a domain ('namespace', or equivalently 'root term') of either biological process, molecular function or cellular component [40]. Note that although all GO terms map to one namespace through subtype 'is_a' relationships, it is possible to map to alternate namespaces through 'part_of' relationships. For each GO term, we extracted the full "pedigree" of the term by finding all possible paths from the GO term to the root term following (i) 'is_a' and 'part_of' relationships, and (ii) by following only 'is_a' relationships. An example pedigree is shown in Additional file 3 Figure S2.

#### Mapping non-gene contigs

Contigs may align with a region of the zebra finch genome but not have significant blast alignments with any of the genes in the zebra finch cDNA database, either because some genes are not yet annotated in zebra finch, or because they are aligning to non-exonic gene features, i.e. introns, 5' untranslated regions (5' UTR) or 3' UTR. It is generally assumed that transcriptome sequencing preferentially sequences mRNA, and that other RNA species such as pre-mRNA, the precursor molecule to mRNA, are either not amplified or are present in such small quantities in the cell that sequencing them is unlikely (although see Bakel *et al*. [41]). To determine whether non-coding contigs represent pre-mRNA, the location of the nearest gene was calculated by finding the shortest distance between the genome location of known zebra finch genes and the position of the contig alignment to the zebra finch genome (that is, the shortest distance to the start of the 5' UTR or the end of the 3' UTR). The contig was classified into one of six classes according to its genome location relative to the closest mapped gene; class A-contig is downstream from the nearest gene, class B-contig is upstream from the gene, class C-contig is downstream but overlapping one end of the gene, class D-contig is upstream but overlapping one end of the gene, class E-the contig spans the gene, class F-the gene spans the contig (Table 5). Note that "upstream" and "downstream" refer to the orientation of the zebra finch genome assembly, for example, position 345 on Tgu1 is upstream from position 350 on Tgu1. Contigs in classes C, D, E and F were further classified to determine whether the 5' and 3' ends of the contig aligned to the 5' UTR, exons, introns or 3' UTR of the gene.

In order to determine whether non-coding contigs were closer to genes than expected by chance, the distribution of contigs relative to genes in the zebra finch genome was assessed by simulating the random placement of contigs onto the zebra finch genome and calculating the minimum distance from the start or end of a contig to the start or end of a gene. Contigs were selected at random, placed at a randomly chosen position in the genome, and the minimum distance to a gene calculated. One million replicates were performed to generate a distribution of the distance between randomly placed contigs and the nearest gene, i.e., the distribution expected if contigs were not cDNA generated from mRNA (i.e., gene sequences) but were random fragments of genomic DNA. The observed and simulated distributions were compared with a Kolmogorov-Smirnov test.

Given that class A and B contigs do not overlap annotated coding regions in the zebra finch genome, these "non-gene" contig sequences may represent non-coding RNA, although it is possible that they instead represent gene sequences which are unannotated in the zebra finch genome, or that the start or end position of genes is imprecise or differs between great tit and zebra finch. To assess the alignment between class A and B contigs with non-coding RNA, non-coding RNA sequences (including miRNA (micro), rRNA (ribosomal), snoRNA (small nucleolar), snRNA (small nuclear) and misc_RNA (miscellaneous)) were downloaded from the Ensembl website http://www.ensembl.org/index.html using the MartView interface of the BioMart data management system http://www.biomart.org. The downloaded sequences were blasted against class A and B contig sequences (blastn with stand-alone BLAST, e-value 10^-5^, all other settings default). The best alignment was retained for each contig.

#### Metagenomics of non-great tit sequences

Some of the assembled contigs may not represent true great tit sequences, for instance if great tit tissue was contaminated with parasites, or RNA from other species was amplified and sequenced due to lab error. Contigs which did not align with zebra finch or chicken genome, cDNA or protein sequences were compared to sequences of other organisms by blasting against the latest NCBI Transcript Reference Sequence RNA database (RefSeq RNA; http://www.ncbi.nlm.nih.gov/RefSeq/) using an e-value of 10^-10 ^and a word size of 20. For each contig, we extracted the genus names of all alignments ("hits") and classified genera as archea, bacteria, algae, protozoa, plant, fungi, invertebrate, vertebrate_fish, vertebrate_amphibians, vertebrate_mammalian, vertebrate_reptiles, vertebrate_birds, unknown or no_hits. Contigs with at least one hit to vertebrate_birds were considered likely to represent true great tit sequences, while contigs which aligned with no vertebrate sequences were further investigated by blasting against sequences from species important in the ecology of the great tit. All available DNA sequences from common great tit ectoparasites (genus *Ceratophyllus *(flea) and *Ixodes ricinus *(tick)), endoparasites (*Plasmodium relictum*, *Trypanosoma everetti*, *Trypanosoma avium *and genera *Haemoproteus*, *Hepatozoon *and *Leucocytozoon*), common trees in Wytham Woods (*Quercus robur *(oak), *Fraxinus excelsior *(ash), *Fagus sylvatica *(beech), *Corylus avellana *(hazel) and *Acer pseudoplatanus *(sycamore)), a common foodsource (*Operophtera brumata *(winter moth)), and species whose fibre/hair is used in great tit nest-building (genus *Poa *(grass), *Meles meles *(badger) and *Ovis aries *(sheep)) were downloaded from NCBI (http://www.ncbi.nlm.nih.gov/nuccore, microsatellites excluded) and saved as a file of 'environmental' sequences. Environmental sequences were blasted against contigs with e-value of 10^-10^. Singleton reads which were not assembled into contigs were also blasted against the 'environmental' sequences, with e-value cut-off of 10^-10^.

#### Microsatellite repeat discovery

Microsatellite markers remain an informative and cost-effective tool for small scale population genetic studies. To detect repeats which may be screened to find polymorphic microsatellite markers in the great tit, a modified version of SPUTNIK (http://wheat.pw.usda.gov/ITMI/EST-SSR/LaRota) was run to find contigs containing microsatellites with five or more di-, tri-, tetra-and penta-nucleotide repeats (program parameters: unit length = 2-5, points for a mismatch = -6, minimum score = 6, adjust scores for the first unit cell). Repeats and their flanking sequences (100 bp) were blasted against passerine microsatellite sequences (downloaded from NCBI; http://www.ncbi.nlm.nih.gov/nuccore, search term 'passeriformes[orgn] AND microsatellite') to determine whether they had already been isolated in other passerines.

#### Single Nucleotide Polymorphism (SNP) discovery

One of the objectives of the transcriptome sequencing was to identify SNPs which could eventually be typed in a large sample of well-phenotyped individuals as part of a QTL mapping experiment. SeqMan NGen was used to produce a summary file of all SNPs and insertion-deletions (indels) in the great tit contig sequences, selecting only those SNPs with base call quality score greater than 30 and where the six neighbouring bases had a quality score more than 20. The summary file was filtered in a hierarchical manner to give a set of SNPs most likely to be segregating in the Wytham Woods population. The summary file was first screened to remove indels, polymorphisms of depth less than eight reads, and polymorphisms of depth 8-20 with minor allele frequency of less than 25%. Polymorphisms were then further screened to produce "high" and "modest" quality SNP files. For the high quality SNPs, (i) polymorphisms of depth greater than 20 with minor allele count of less than five were removed and (ii) polymorphisms located less than 50 bases from another polymorphism or from the start or end of a contig were removed. For the modest quality SNPs, (i) polymorphisms of depth greater than 20 with minor allele count of less than three were removed and (ii) polymorphisms located less than 40 bases from another polymorphism or from the start or end of a contig were removed. The majority of the high quality SNPs were reported in the modest quality file, although some previously detected high quality SNPs were excluded from the modest quality file because the inclusion of lower stringency SNPs meant they now mapped within 40 bases of another SNP.

The SNP discovery process was then repeated by reassembling the reads onto the great tit assembly using a higher match percentage (90, default 85) than the default settings ('remap assembly'). The match size was kept at the default value (25) rather than increased to 41 (as previously) to allow some movement of sequences from the contigs they were initially assembled to. Any SNPs from the first discovery phase that were not detected in the remap assembly were excluded from further analysis, as they are not robust to small changes in assembly parameters, and hence may not be true polymorphisms.

#### Alternative splicing

Alternative splicing produces genetic variation without the need for additional genes [33,42]. However, genes which are alternatively spliced are a challenge to the assembly of transcriptome sequence into contigs, as it is difficult to predict whether reads extending over alternative splice sites will be assembled into the same contig, or split into two or more different contigs (Figure 2b). For the scenario where reads from alternative splice variants are assembled into a single contig, some reads will "jump" one or more exons, resulting in a region of very poor alignment to the consensus sequence. Across many aligned reads, these regions of poor sequence similarity tend to produce a 'string' of putative (probably false) single nucleotide polymorphisms in close proximity. SNPs were extracted from the original unfiltered SNP summary file, and two or more SNPs were defined as being part of a 'string' provided the distance between them was less than eight bases (for example, a SNP string of five could at most be spread over 33 bases). Strings of six or more SNPs were considered likely to represent splice variants.

Unless otherwise stated, all analyses and data management (blast summaries, GO mapping, tissue specificity, alternative splice sites, SNP summaries) were coded in Visual Basic and all graphs produced in R v2.11.

## Authors' contributions

AWS carried out the assembly and transcriptome analysis and drafted the manuscript. JG and JAM prepared the great tit samples and provided comments on the manuscript. BCS provided the great tit samples and provided comments on the manuscript. JS conceived of the study, participated in the design of the analysis and helped to draft the manuscript. All authors read and approved the final manuscript.

## Supplementary Material

Additional file 1 **Table S1**. Contigs with high coverage aligning with zebra finch or chicken genes. an excel table listing great tit contigs with high sequencing coverage which align with zebra finch and/or chicken genes. **Table S2**. Unique GO terms associated with contigs expressed in only one tissue. an excel table listing the unique GO terms associated with contigs expressed only in one tissue. **Table S3**. Classification of unique GO terms into children of the three namespaces. an excel table listing the classification of all the GO terms uniquely associated with contigs expressed in one (or all) tissues into the 65 GO terms which are 'children' of the three namespaces (molecular function, biological process and cellular component). **Table S4**. Microsatellites detected in the great tit transcriptome. an excel table listing repeats detected in the great tit assembly. Repeats are listed along with at least 10 bases of flanking sequence on each side (up to a maximum of 200 bases of flanking sequence) Click here for file

Additional file 2 **Figure S1**. Distribution of zebra finch genes across the zebra finch genome. Counts are plotted within 500,000 base pair bins. No genes map to Tgu16 and one gene maps to TguLG5 (not shown). Note that Tgu-22, 25, 26 and 27 are presented out of numeric sequence at the bottom of the plot, to make visual comparison with Figures 3 and 4 easier. an encapsulated postscript (eps) file showing the distribution of zebra finch genes across the zebra finch genome Click here for file

Additional file 3 **Figure S2**. Example 'is_a' and 'part_of' pedigree mapping of the GO term GO:0022857 (name: transmembrane transporter activity). The 'is_a'-only mapping is shown light grey; GO:0022857 is a subtype of GO:0005215 (transporter activity), which is a subtype of the root term GO:0003674 (molecular function), while GO:0022857 can also be mapped to the root term GO:0008150 (biological process) through 'part_of' relationships. a powerpoint (ppt) file with an example pedigree mapping of the GO term GO:0022857 to its 'parent' terms through "is_a" and "part_of" relationships Click here for file
