# Supplementary figures and images for: Characterisation of the transcriptome of a wild great tit Parus major population by next generation sequencing
Source: BMC Genomics. 2011 Jun 2;12:283. doi: 10.1186/1471-2164-12-283 (PMC3125266; doi:10.1186/1471-2164-12-283)

## Slide 1
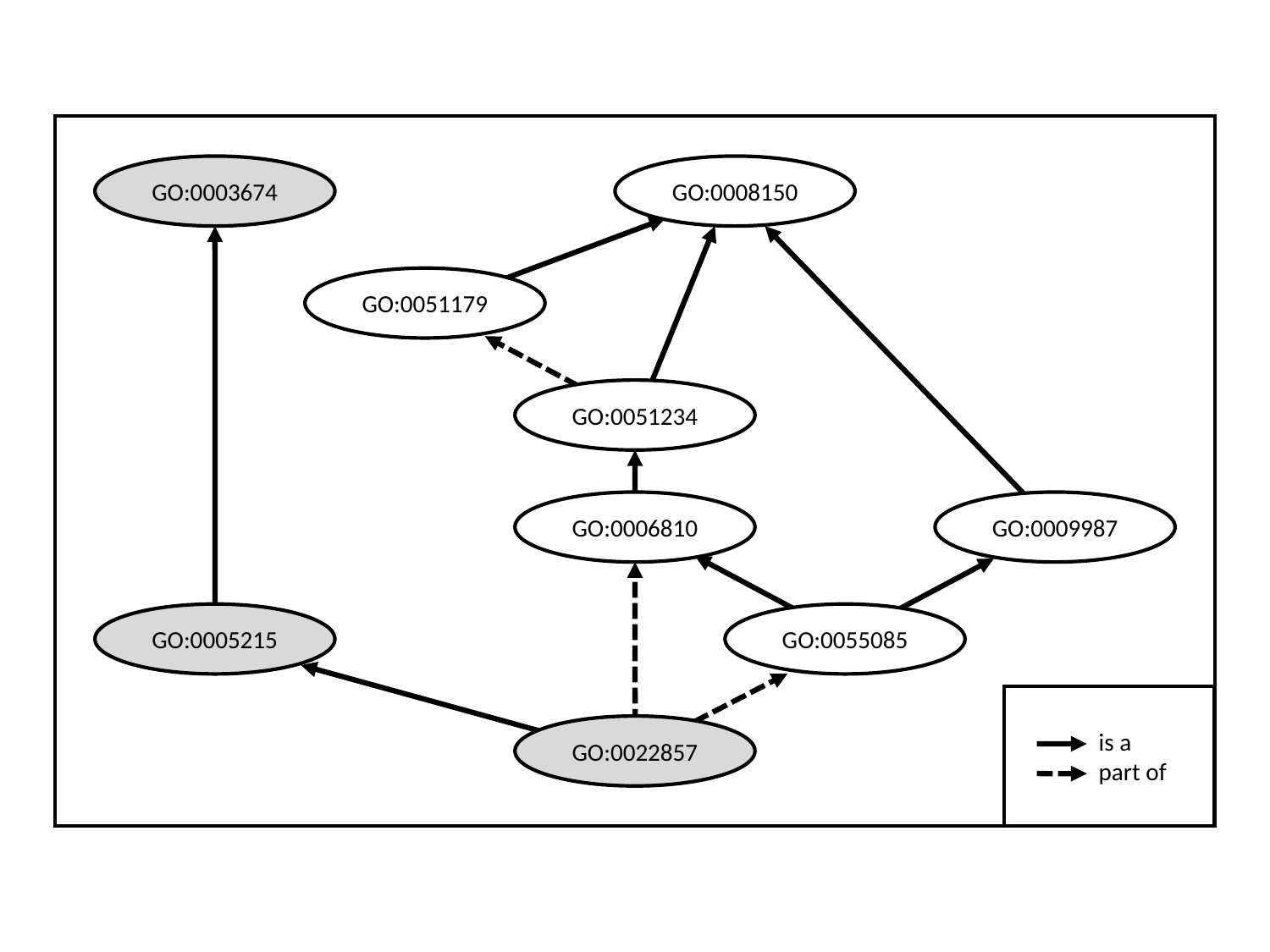

GO:0003674
GO:0008150
GO:0051179
GO:0051234
GO:0006810
GO:0009987
GO:0005215
GO:0055085
GO:0022857
is a
part of

Supplement: Additional file 3 — Figure S2. Example 'is_a' and 'part_of' pedigree mapping of the GO term GO:0022857 (name: transmembrane transporter activity). The 'is_a'-only mapping is shown light grey; GO:0022857 is a subtype of GO:0005215 (transporter activity), which is a subtype of the root term GO:0003674 (molecular function), while GO:0022857 can also be mapped to the root term GO:0008150 (biological process) through 'part_of' relationships. a powerpoint (ppt) file with an example pedigree mapping of the GO term GO:0022857 to its 'parent' terms through "is_a" and "part_of" relationships [file 1471-2164-12-283-S3.PPT]
